# 4-Meth­oxy­benzamidinium hydrogen sulfate

**DOI:** 10.1107/S1600536812044327

**Published:** 2012-10-31

**Authors:** Simona Irrera, Gustavo Portalone

**Affiliations:** a Chemistry Department, ‘Sapienza’ University of Rome, P. le A. Moro, 5, I-00185 Rome, Italy

## Abstract

The title salt, C_8_H_11_N_2_O^+^·HSO_4_^−^, has been synthesized by the reaction between 4-meth­oxy­benzamidine and sulfuric acid. The asymmetric unit comprises a nonplanar 4-meth­oxy­benzamidinium cation and one hydrogen sulfate anion. In the cation, the amidinium group has two identical C—N bonds [1.306 (2) and 1.308 (2) Å], and its plane forms a dihedral angle of 6.49 (8)° with the mean plane of the benzene ring. The ionic components are associated in the crystal *via* N—H^+^⋯O^−^, resulting in chains running approximately along the *b*-axis direction whicg are interconnected by O—H⋯O^−^ hydrogen bonds.

## Related literature

For the biological and pharmacological relevance of benzamidine, see: Powers & Harper (1999 ▶); Grzesiak *et al.* (2000 ▶). For structural analysis of proton-transfer adducts containing mol­ecules of biological inter­est, see: Portalone (2011*a* ▶); Portalone & Irrera (2011 ▶). For the supra­molecular association in proton-transfer adducts containing benzamidinium cations, see: Portalone (2010 ▶, 2011*b* ▶, 2012 ▶); Irrera & Portalone (2012 ▶); Irrera *et al.* (2012 ▶). For hydrogen-bond motifs, see Bernstein *et al.* (1995 ▶).
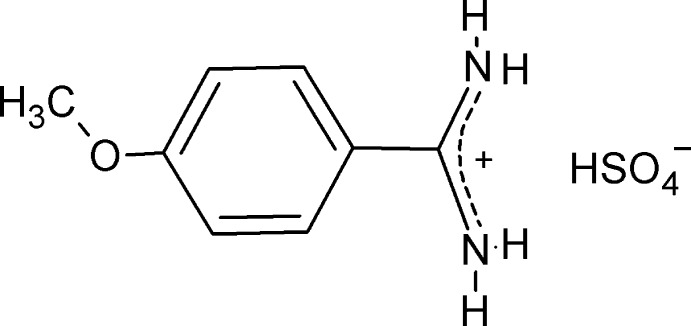


## Experimental

### 

#### Crystal data

C_8_H_11_N_2_O^+^·HSO_4_^−^
*M* *_r_* = 248.26
Monoclinic, 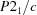

*a* = 14.2608 (14) Å
*b* = 10.1844 (9) Å
*c* = 7.5723 (9) Å
β = 94.206 (10)°
*V* = 1096.83 (19) Å^3^
*Z* = 4
Mo *K*α radiation
μ = 0.30 mm^−1^
*T* = 298 K
0.31 × 0.25 × 0.15 mm

#### Data collection

Oxford Diffraction Xcalibur S CCD diffractometer
Absorption correction: multi-scan (*CrysAlis RED*; Oxford Diffraction, 2006 ▶) *T*_min_ = 0.912, *T*_max_ = 0.956
16529 measured reflections
2626 independent reflections
2133 reflections with *I* > 2σ(*I*)
*R*_int_ = 0.036

#### Refinement

*R*[*F* ^2^ > 2σ(*F* ^2^)] = 0.039
*wR*(*F* ^2^) = 0.114
*S* = 1.04
2626 reflections
167 parameters
H atoms treated by a mixture of independent and constrained refinement
Δρ_max_ = 0.26 e Å^−3^
Δρ_min_ = −0.34 e Å^−3^

### 

Data collection: *CrysAlis CCD* (Oxford Diffraction, 2006 ▶); cell refinement: *CrysAlis RED* (Oxford Diffraction, 2006 ▶); data reduction: *CrysAlis RED*; program(s) used to solve structure: *SIR97* (Altomare *et al.*, 1999 ▶); program(s) used to refine structure: *SHELXL97* (Sheldrick, 2008 ▶); molecular graphics: *ORTEP-3* (Farrugia, 1997 ▶); software used to prepare material for publication: *WinGX* (Farrugia, 1999 ▶).

## Supplementary Material

Crystal structure: contains datablock(s) global, I. DOI: 10.1107/S1600536812044327/rz5020sup1.cif

Structure factors: contains datablock(s) I. DOI: 10.1107/S1600536812044327/rz5020Isup2.hkl

Additional supplementary materials:  crystallographic information; 3D view; checkCIF report

## Figures and Tables

**Table 1:** Hydrogen-bond geometry (Å, °)

*D*—H⋯*A*	*D*—H	H⋯*A*	*D*⋯*A*	*D*—H⋯*A*
O3—H3*A*⋯O2^i^	0.78 (3)	1.79 (3)	2.562 (2)	172 (3)
N1—H1*A*⋯O4	0.86 (2)	2.10 (2)	2.938 (2)	163 (2)
N1—H1*B*⋯O5^ii^	0.84 (2)	2.10 (2)	2.884 (2)	154 (2)
N2—H2*A*⋯O5	0.84 (2)	2.07 (3)	2.907 (2)	177 (2)
N2—H2*B*⋯O4^iii^	0.86 (3)	2.22 (3)	2.965 (2)	145 (3)

Symmetry codes: (i) 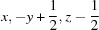; (ii) 
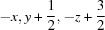
; (iii) 
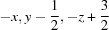
.

**Fig. 1.**
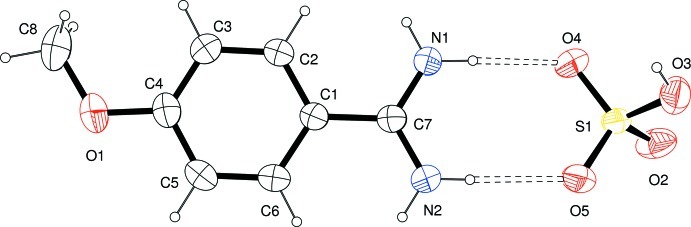
The asymmetric unit of (I), showing displacements ellipsoids drawn at the 50% probability level. The asymmetric unit was selected so that the two ions are linked by N+.—H···O- hydrogen bonds. H atoms are shown as small spheres of arbitrary radii. Hydrogen bonding is indicated by dashed lines.

**Fig. 2.**
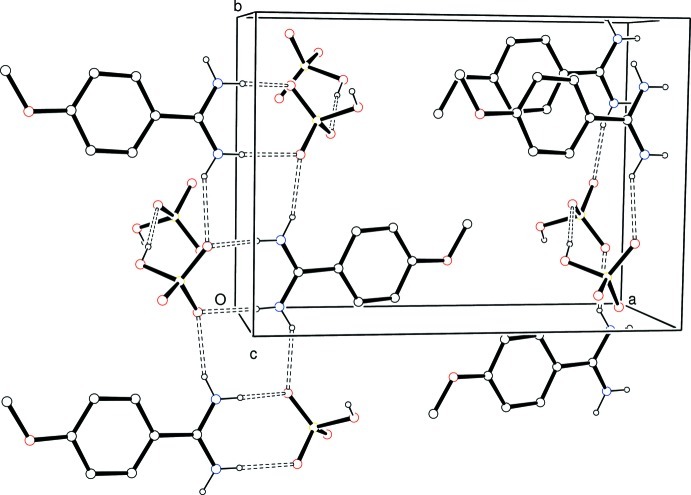
Crystal packing diagram for (I), viewed approximately down the c axis. Displacements ellipsoids are at the 50% probability level. H atoms are shown as small spheres of arbitrary radii. For the sake of clarity, H atoms not involved in hydrogen bonding (dashed lines) have been omitted.

## supplementary crystallographic information

### Comment

In our search for new salt compounds as part of investigation of the functionality of biological systems (Portalone, 2011*a*; Portalone & Irrera, 2011), we have prepared 4-methoxybenzamidinium hydrogen sulfate, (I), which was obtained by a reaction between 4-methoxybenzamidine (4-amidinoanisole) and sulfuric acid.

Benzamidine derivatives, which have shown strong biological and pharmacological activity (Powers & Harper, 1999; Grzesiak *et al.*, 2000), are being used in our group as bricks for supramolecular construction (Portalone, 2010, 2011*b*, 2012). Indeed, these molecules are strong Lewis bases and their cations can be easily anchored onto numerous inorganic and organic anions and polyanions, largely because of the presence of four potential donor sites for hydrogen-bonding.

The asymmetric unit of (I) comprises one non-planar 4-methoxybenzamidinium cation and one hydrogen sulfate anion (Fig. 1). In the cation the amidinium group forms dihedral angle of 6.49 (8)° with the mean plane of the phenyl ring, which is close to the the values observed in protonated benzamidinium ions (14.4 (1) - 32.7 (1)°, Portalone, 2010, 2012; Irrera *et al.*, 2012). The lack of planarity in all these systems is obviously caused by steric hindrances between the H atoms of the aromatic ring and the amidine moiety. This conformation is rather common in benzamidinium-containing small-molecule crystal structures, with the only exception of benzamidinium diliturate, where the benzamidinium cation is planar (Portalone, 2010). The pattern of bond lengths and bond angles of the 4-methoxybenzamidinium cation agrees with that reported in previous structural investigations (Irrera *et al.*, 2012; Portalone, 2010, 2012; Irrera & Portalone, 2012). In particular the amidinium group, true to one's expectations, features identical C—N bonds within experimental error [1.306 (2) and 1.308 (2) Å], evidencing the delocalization of the π electrons and double-bond character.

Bond lengths in the slightly distorted tetrahedral hydrogen sulfate anion indicate the position of the H atom. There are three short S—O bonds of 1.4504 (13), 1.4442 (13) and 1.4464 (13) Å to terminal atoms O2, O4 and O5, respectively, and one longer bond of 1.5470 (15) Å to atom O3, which is bound to atom H3A.

The ionic components of compound (I) are joined by two N^+^—H···O^-^ (±) hydrogen bonds (Table 1) to form ionic dimers with graph-set motif *R*^2^_2_(8) (Bernstein *et al.*, 1995). Analysis of the crystal packing of (I), (Fig. 2), shows that four N^+^—H···O^-^ hydrogen bonds link the molecular components into a mono-dimensional structure. As previously mentioned, each subunit, built from the ion pairs of the asymmetric unit, forms *R*^2^_2_(8) dimers *via* the bidentate interaction of the N—H and S—O groups. Adjacent ion pairs are then linked together by way of the remaining two N^+^—H···O^-^ hydrogen bonds to form *R*^2^_4_(8), resulting in chains running approximately along crystallographic *b* axis. These chains are then interconnected by means of the only O—H···O hydrogen bond present in the structure.

### Experimental

4-Methoxybenzamidine (0.1 mmol, Fluka at 96% purity) was dissolved without further purification in 6 ml of hot water and heated under reflux for 3 h. While stirring, H_2_SO_4_ (2 mol *L*^-1^) was added dropwise until pH reached 2. After cooling the solution to an ambient temperature, colourless crystals suitable for single-crystal X-ray diffraction were grown by slow evaporation of the solvent after one week.

### Refinement

All H atoms were identified in difference Fourier maps, but for refinement all C-bound H atoms were placed in calculated positions, with C—H = 0.93 Å (phenyl) and 0.97 Å (methyl), and refined as riding on their carrier atoms. The *U*_iso_ values were kept equal to 1.2*U*_eq_(C, phenyl). and to 1.5*U*_eq_(C, methyl). Positional and thermal parameters of H atoms of the amidinium group and of the hydrogen sulfate ion were freely refined, giving N—H distances in the range 0.84 (2)–0.86 (2) Å and an O—H distance equal to 0.78 (3) Å.

### Crystal data

**Table:**

C_8_H_11_N_2_O^+^·HSO_4_^−^	*F*(000) = 520
*M**_r_* = 248.26	*D*_x_ = 1.503 Mg m^−^^3^
Monoclinic, *P*2_1_/*c*	Mo *K*α radiation, λ = 0.71069 Å
Hall symbol: -P 2ybc	Cell parameters from 4647 reflections
*a* = 14.2608 (14) Å	θ = 2.9–29.7°
*b* = 10.1844 (9) Å	µ = 0.30 mm^−^^1^
*c* = 7.5723 (9) Å	*T* = 298 K
β = 94.206 (10)°	Tablets, colourless
*V* = 1096.83 (19) Å^3^	0.31 × 0.25 × 0.15 mm
*Z* = 4	

### Data collection

**Table:**

Oxford Diffraction Xcalibur S CCD diffractometer	2626 independent reflections
Radiation source: Enhance (Mo) X-ray Source	2133 reflections with *I* > 2σ(*I*)
Graphite monochromator	*R*_int_ = 0.036
Detector resolution: 16.0696 pixels mm^-1^	θ_max_ = 28.0°, θ_min_ = 2.9°
ω and φ scans	*h* = −18→18
Absorption correction: multi-scan (*CrysAlis RED*; Oxford Diffraction, 2006)	*k* = −13→13
*T*_min_ = 0.912, *T*_max_ = 0.956	*l* = −9→9
16529 measured reflections	

### Refinement

**Table:**

Refinement on *F*^2^	Primary atom site location: structure-invariant direct methods
Least-squares matrix: full	Secondary atom site location: difference Fourier map
*R*[*F*^2^ > 2σ(*F*^2^)] = 0.039	Hydrogen site location: inferred from neighbouring sites
*wR*(*F*^2^) = 0.114	H atoms treated by a mixture of independent and constrained refinement
*S* = 1.04	*w* = 1/[σ^2^(*F*_o_^2^) + (0.0582*P*)^2^ + 0.2957*P*] where *P* = (*F*_o_^2^ + 2*F*_c_^2^)/3
2626 reflections	(Δ/σ)_max_ < 0.001
167 parameters	Δρ_max_ = 0.26 e Å^−^^3^
0 restraints	Δρ_min_ = −0.34 e Å^−^^3^

### Special details

**Table:**

Experimental. Absorption correction: [CrysAlis RED (Oxford Diffraction, 2006); empirical absorption correction using spherical harmonics, implemented in SCALE3 ABSPACK scaling algorithm]
Geometry. All s.u.'s (except the s.u. in the dihedral angle between two l.s. planes) are estimated using the full covariance matrix. The cell s.u.'s are taken into account individually in the estimation of s.u.'s in distances, angles and torsion angles; correlations between s.u.'s in cell parameters are only used when they are defined by crystal symmetry. An approximate (isotropic) treatment of cell s.u.'s is used for estimating s.u.'s involving l.s. planes.
Refinement. Refinement of *F*^2^ against ALL reflections. The weighted *R*-factor *wR* and goodness of fit *S* are based on *F*^2^, conventional *R*-factors *R* are based on *F*, with *F* set to zero for negative *F*^2^. The threshold expression of *F*^2^ > 2σ(*F*^2^) is used only for calculating *R*-factors(gt) *etc*. and is not relevant to the choice of reflections for refinement. *R*-factors based on *F*^2^ are statistically about twice as large as those based on *F*, and *R*- factors based on ALL data will be even larger.

### Fractional atomic coordinates and isotropic or equivalent isotropic displacement parameters (Å^2^)

**Table:**

	*x*	*y*	*z*	*U*_iso_*/*U*_eq_	
S1	−0.16129 (3)	0.16203 (4)	0.84884 (6)	0.03448 (15)	
O2	−0.20315 (10)	0.11649 (13)	1.00664 (17)	0.0514 (4)	
O3	−0.24735 (9)	0.20809 (15)	0.7284 (2)	0.0498 (4)	
H3A	−0.2287 (18)	0.259 (3)	0.662 (4)	0.064 (8)*	
O4	−0.09972 (9)	0.27365 (12)	0.87891 (18)	0.0467 (3)	
O5	−0.11706 (9)	0.05608 (12)	0.75864 (19)	0.0485 (3)	
O1	0.49301 (10)	0.19362 (16)	0.4667 (3)	0.0684 (5)	
N1	0.07593 (12)	0.28008 (15)	0.6992 (3)	0.0452 (4)	
H1A	0.0189 (18)	0.280 (2)	0.730 (3)	0.053 (6)*	
H1B	0.1027 (17)	0.354 (2)	0.693 (3)	0.061 (7)*	
N2	0.07598 (14)	0.06125 (17)	0.6593 (3)	0.0595 (5)	
H2A	0.0212 (18)	0.058 (2)	0.692 (3)	0.057 (7)*	
H2B	0.107 (2)	−0.009 (3)	0.647 (4)	0.087 (9)*	
C1	0.21780 (12)	0.18063 (15)	0.6067 (2)	0.0346 (4)	
C2	0.26115 (13)	0.30028 (17)	0.5855 (3)	0.0465 (5)	
H2	0.2275	0.3770	0.6020	0.056*	
C3	0.35275 (14)	0.30917 (18)	0.5407 (3)	0.0497 (5)	
H3	0.3806	0.3910	0.5288	0.060*	
C4	0.40279 (13)	0.19663 (19)	0.5136 (3)	0.0465 (5)	
C5	0.36028 (15)	0.0754 (2)	0.5315 (3)	0.0595 (6)	
H5	0.3937	−0.0010	0.5123	0.071*	
C6	0.26906 (14)	0.06751 (18)	0.5775 (3)	0.0493 (5)	
H6	0.2413	−0.0143	0.5892	0.059*	
C7	0.12029 (12)	0.17365 (15)	0.6575 (2)	0.0365 (4)	
C8	0.54205 (16)	0.3152 (3)	0.4575 (4)	0.0749 (8)	
H8A	0.5094 (10)	0.3716 (13)	0.369 (2)	0.112*	
H8B	0.6058 (12)	0.2988 (4)	0.425 (3)	0.112*	
H8C	0.5445 (13)	0.3583 (13)	0.573 (2)	0.112*	

### Atomic displacement parameters (Å^2^)

**Table:**

	*U*^11^	*U*^22^	*U*^33^	*U*^12^	*U*^13^	*U*^23^
S1	0.0360 (3)	0.0244 (2)	0.0440 (3)	−0.00176 (15)	0.00969 (18)	−0.00018 (16)
O2	0.0718 (9)	0.0368 (7)	0.0477 (8)	−0.0093 (6)	0.0198 (7)	0.0026 (6)
O3	0.0352 (7)	0.0531 (8)	0.0616 (9)	−0.0021 (6)	0.0082 (6)	0.0112 (7)
O4	0.0456 (7)	0.0321 (7)	0.0627 (9)	−0.0101 (5)	0.0068 (6)	−0.0029 (6)
O5	0.0488 (7)	0.0302 (6)	0.0688 (9)	0.0022 (5)	0.0193 (6)	−0.0041 (6)
O1	0.0351 (8)	0.0586 (9)	0.1145 (14)	0.0038 (7)	0.0259 (8)	−0.0054 (9)
N1	0.0333 (8)	0.0306 (8)	0.0735 (12)	−0.0025 (6)	0.0154 (8)	−0.0090 (7)
N2	0.0389 (10)	0.0285 (8)	0.1137 (17)	−0.0032 (7)	0.0232 (10)	−0.0039 (9)
C1	0.0304 (8)	0.0282 (8)	0.0454 (9)	0.0011 (6)	0.0031 (7)	−0.0015 (7)
C2	0.0367 (9)	0.0277 (8)	0.0763 (14)	0.0020 (7)	0.0133 (9)	−0.0030 (8)
C3	0.0395 (10)	0.0335 (9)	0.0775 (14)	−0.0050 (8)	0.0137 (9)	−0.0031 (9)
C4	0.0309 (9)	0.0457 (10)	0.0639 (12)	0.0020 (8)	0.0097 (8)	−0.0032 (9)
C5	0.0448 (11)	0.0352 (10)	0.1007 (17)	0.0089 (8)	0.0210 (11)	−0.0056 (10)
C6	0.0423 (10)	0.0277 (8)	0.0793 (14)	0.0008 (7)	0.0132 (9)	−0.0048 (9)
C7	0.0328 (9)	0.0283 (8)	0.0483 (10)	−0.0003 (6)	0.0023 (7)	−0.0020 (7)
C8	0.0381 (12)	0.0773 (17)	0.112 (2)	−0.0104 (11)	0.0238 (13)	−0.0012 (15)

### Geometric parameters (Å, º)

**Table:**

S1—O4	1.4442 (13)	C1—C6	1.391 (2)
S1—O5	1.4464 (13)	C1—C7	1.471 (2)
S1—O2	1.4504 (13)	C2—C3	1.377 (3)
S1—O3	1.5470 (15)	C2—H2	0.9300
O3—H3A	0.78 (3)	C3—C4	1.374 (3)
O1—C4	1.360 (2)	C3—H3	0.9300
O1—C8	1.427 (3)	C4—C5	1.386 (3)
N1—C7	1.306 (2)	C5—C6	1.374 (3)
N1—H1A	0.86 (2)	C5—H5	0.9300
N1—H1B	0.84 (2)	C6—H6	0.9300
N2—C7	1.308 (2)	C8—H8A	0.9739
N2—H2A	0.84 (2)	C8—H8B	0.9739
N2—H2B	0.86 (3)	C8—H8C	0.9739
C1—C2	1.381 (2)		
			
O4—S1—O5	112.37 (8)	C4—C3—H3	120.2
O4—S1—O2	113.83 (8)	C2—C3—H3	120.2
O5—S1—O2	111.75 (8)	O1—C4—C3	124.73 (18)
O4—S1—O3	107.52 (8)	O1—C4—C5	115.75 (17)
O5—S1—O3	107.60 (8)	C3—C4—C5	119.52 (17)
O2—S1—O3	103.05 (9)	C6—C5—C4	120.42 (17)
S1—O3—H3A	106.6 (19)	C6—C5—H5	119.8
C4—O1—C8	118.01 (17)	C4—C5—H5	119.8
C7—N1—H1A	123.0 (15)	C5—C6—C1	120.68 (17)
C7—N1—H1B	119.6 (16)	C5—C6—H6	119.7
H1A—N1—H1B	117 (2)	C1—C6—H6	119.7
C7—N2—H2A	120.2 (16)	N1—C7—N2	118.74 (18)
C7—N2—H2B	118.6 (19)	N1—C7—C1	120.48 (15)
H2A—N2—H2B	120 (2)	N2—C7—C1	120.77 (16)
C2—C1—C6	117.86 (16)	O1—C8—H8A	109.5
C2—C1—C7	120.84 (15)	O1—C8—H8B	109.5
C6—C1—C7	121.29 (15)	H8A—C8—H8B	109.5
C3—C2—C1	121.85 (16)	O1—C8—H8C	109.5
C3—C2—H2	119.1	H8A—C8—H8C	109.5
C1—C2—H2	119.1	H8B—C8—H8C	109.5
C4—C3—C2	119.65 (17)		
			
C6—C1—C2—C3	−1.4 (3)	C3—C4—C5—C6	−0.5 (4)
C7—C1—C2—C3	179.01 (19)	C4—C5—C6—C1	0.0 (4)
C1—C2—C3—C4	0.9 (3)	C2—C1—C6—C5	0.9 (3)
C8—O1—C4—C3	4.9 (3)	C7—C1—C6—C5	−179.5 (2)
C8—O1—C4—C5	−176.0 (2)	C2—C1—C7—N1	−6.3 (3)
C2—C3—C4—O1	179.1 (2)	C6—C1—C7—N1	174.06 (19)
C2—C3—C4—C5	0.1 (3)	C2—C1—C7—N2	172.7 (2)
O1—C4—C5—C6	−179.6 (2)	C6—C1—C7—N2	−6.9 (3)

### Hydrogen-bond geometry (Å, º)

**Table:**

*D*—H···*A*	*D*—H	H···*A*	*D*···*A*	*D*—H···*A*
O3—H3*A*···O2^i^	0.78 (3)	1.79 (3)	2.562 (2)	172 (3)
N1—H1*A*···O4	0.86 (2)	2.10 (2)	2.938 (2)	163 (2)
N1—H1*B*···O5^ii^	0.84 (2)	2.10 (2)	2.884 (2)	154 (2)
N2—H2*A*···O5	0.84 (2)	2.07 (3)	2.907 (2)	177 (2)
N2—H2*B*···O4^iii^	0.86 (3)	2.22 (3)	2.965 (2)	145 (3)

Symmetry codes: (i) *x*, −*y*+1/2, *z*−1/2; (ii) −*x*, *y*+1/2, −*z*+3/2; (iii) −*x*, *y*−1/2, −*z*+3/2.

## References

[bb1] Altomare, A., Burla, M. C., Camalli, M., Cascarano, G. L., Giacovazzo, C., Guagliardi, A., Moliterni, A. G. G., Polidori, G. & Spagna, R. (1999). *J. Appl. Cryst.* **32**, 115–119.

[bb2] Bernstein, J., Davis, R. E., Shimoni, L. & Chang, N.-L. (1995). *Angew. Chem. Int. Ed. Engl.* **34**, 1555–1573.

[bb3] Farrugia, L. J. (1997). *J. Appl. Cryst.* **30**, 565.

[bb4] Farrugia, L. J. (1999). *J. Appl. Cryst.* **32**, 837–838.

[bb5] Grzesiak, A., Helland, R., Smalas, A. O., Krowarsch, D., Dadlez, M. & Otlewski, J. (2000). *J. Mol. Biol.* **301**, 205–217. 10.1006/jmbi.2000.3935 10926503

[bb6] Irrera, S., Ortaggi, G. & Portalone, G. (2012). *Acta Cryst.* C**68**, o447–o451. 10.1107/S010827011204067X 23124460

[bb7] Irrera, S. & Portalone, G. (2012). *Acta Cryst.* E**68**, o3083. 10.1107/S1600536812041219 PMC3515188 23284415

[bb8] Oxford Diffraction (2006). *CrysAlis CCD* and *CrysAlis RED* Oxford Diffraction Ltd, Yarnton, Oxfordshire, England.

[bb9] Portalone, G. (2010). *Acta Cryst.* C**66**, o295–o301. 10.1107/S0108270110016252 20522949

[bb10] Portalone, G. (2011*a*). *Chem. Cent. J.* **5**, 51. 10.1186/1752-153X-5-51 PMC3182958 21888640

[bb11] Portalone, G. (2011*b*). *Acta Cryst.* E**67**, o3394–o3395. 10.1107/S1600536811049075 PMC3239035 22199883

[bb12] Portalone, G. (2012). *Acta Cryst.* E**68**, o268–o269. 10.1107/S160053681105519X PMC3274967 22346912

[bb13] Portalone, G. & Irrera, S. (2011). *J. Mol. Struct.* **991**, 92–96.

[bb14] Powers, J. C. & Harper, J. W. (1999). *Proteinase Inhibitors*, edited by A. J. Barrett & G. Salvesen, pp. 55–152. Amsterdam: Elsevier.

[bb15] Sheldrick, G. M. (2008). *Acta Cryst.* A**64**, 112–122. 10.1107/S0108767307043930 18156677

